# HIV Associated Risk Factors for Ischemic Stroke and Future Perspectives

**DOI:** 10.3390/ijms21155306

**Published:** 2020-07-26

**Authors:** Saifudeen Ismael, Mohammad Moshahid Khan, Prashant Kumar, Sunitha Kodidela, Golnoush Mirzahosseini, Santhosh Kumar, Tauheed Ishrat

**Affiliations:** 1 Department of Anatomy and Neurobiology, University of Tennessee Health Science Center, Memphis, TN 38163, USA; sismael@uthsc.edu (S.I.); gmirzaho@uthsc.edu (G.M.); 2 Department of Neurology, University of Tennessee Health Science Center, Memphis, TN 38163, USA; mkhan26@uthsc.edu; 3 Division of Rehabilitation Sciences and Department of Physical Therapy, College of Health Professions, University of Tennessee Health Science Center, Memphis, TN 38163, USA; 4 Department of Pediatrics, University of Tennessee Health Science Center and Le Bonheur Children’s Hospital, Memphis, TN 38163, USA; pkumar21@uthsc.edu; 5 Department of Pharmaceutical Sciences, The University of Tennessee Health Science Center, Memphis, TN 38163, USA; skodidel@uthsc.edu (S.K.); ksantosh@uthsc.edu (S.K.); 6 Neuroscience Institute, University of Tennessee Health Science Center, Memphis, TN 38163, USA

**Keywords:** ischemic stroke, HIV, extracellular vesicles, glycoprotein-120, trans-activator of transcription

## Abstract

Although retroviral therapy (ART) has changed the HIV infection from a fatal event to a chronic disease, treated HIV patients demonstrate high prevalence of HIV associated comorbidities including cardio/cerebrovascular diseases. The incidence of stroke in HIV infected subjects is three times higher than that of uninfected controls. Several clinical and postmortem studies have documented the higher incidence of ischemic stroke in HIV infected patients. The etiology of stroke in HIV infected patients remains unknown; however, several factors such as coagulopathies, opportunistic infections, vascular abnormalities, atherosclerosis and diabetes can contribute to the pathogenesis of stroke. In addition, chronic administration of ART contributes to the increased risk of stroke in HIV infected patients. Concurrently, experimental studies in murine model of ischemic stroke demonstrated that HIV infection worsens stroke outcome, increases blood brain barrier permeability and increases neuroinflammation. Additionally, residual HIV viral proteins, such as Trans-Activator of Transcription, glycoprotein 120 and Negative regulatory factor, contribute to the pathogenesis. This review presents comprehensive information detailing the risk factors contributing to ischemic stroke in HIV infected patients. It also outlines experimental evidence demonstrating the impact of HIV infection on stroke outcomes, in addition to possible novel therapeutic approaches to improve these outcomes.

## 1. Introduction

Human immunodeficiency virus (HIV) infection causes a progressive depletion of the immune system, leading to acquired immunodeficiency syndrome (AIDS). An estimated 37.9 million people worldwide are infected with HIV, and tens of millions of people have died of AIDS-related complications since the beginning of the epidemic. While there is no cure for HIV infection, antiretroviral therapy (ART) has changed this from a fatal disease to a chronic, and manageable condition for people living with HIV (PLWH) [1,2]. However, despite treatment HIV infected patients still have compromised health, which has led to a higher prevalence of HIV-associated comorbidities, especially neuronal disorders, compared to those without HIV infection [3,4].

Noninfectious comorbidities have been considered an important source of functional impairment and mortality in HIV-infected populations, with cerebrovascular disease being the leading cause of death [5,6,7,8,9]. Stroke is an abrupt interruption of constant blood supply to the brain, causing permanent or partial loss of brain function. Several clinical and postmortem studies have documented the association between HIV infection and the risk of stroke [6,10,11,12,13,14]. The number of stroke hospitalizations in the HIV infected population has dramatically increased in recent years, despite an overall decline in the number of stroke hospitalizations alone [7]. The increased risk of stroke in HIV infected subjects is not known, but several factors, including opportunistic infections, aging, male/female gender, tumors, atherosclerosis, diabetes, hypertension, autoimmunity, vascular abnormalities, coagulopathies, and cardiovascular disease influence the risk of stroke in HIV infected patients [15,16,17,18,19,20]. In addition, chronic exposure to ART contributes to the risk of stroke in HIV infected patients, both directly by accelerating atherosclerosis and indirectly by enhancing longevity [10,21].

Stroke was first reported in patients with HIV infection by Anders and colleagues [22]. The number of stroke incidents were noted to be higher in HIV-infected children and young adults, without traditional risk factors [23,24]. These strokes were mainly ischemic in nature and often went unnoticed and undiagnosed throughout a person’s lifetime. In the pre-ART era, PLWH had a high risk of ischemic stroke compared to those without HIV and the strokes occurred mostly in those with advanced AIDS, complicated with secondary infections such as toxoplasmosis encephalitis, fungal meningitis, tuberculous meningitis and neurosyphilis or those with coagulopathies or vasculitis [25,26,27]. A pre-ART population-based study by Cole and coworkers documented that the incidence of ischemic stroke was nine times higher in AIDS patients than in control individuals [28]]. Similarly, Engstrom et al. reported an increased risk of stroke in patients with AIDS compared to the reference population [29]. In contrast, a retrospective case-controlled study by Hoffmann et al. reported no increase in the incidence of stroke in HIV-infected patients compared to age-matched controls [30]. However, in the post-ART era, several studies demonstrated an increased risk of stroke in PLWH independent of age and traditional vascular risk factors [31,32,33]. Subsai and coworkers demonstrated that, post-ART, the incidence of ischemic and hemorrhagic stroke was increased in a Thai population infected with HIV [34]. In a large US healthcare insurance database, the incidence of a stroke was approximately three times higher in PLWH than uninfected controls after adjustment for sex and age [12].

Genetic factors also influence the increased risk of stroke in HIV infected patients. Studies have shown an increased risk of stroke in African Americans in an ART-treated PLWH compared with other race/ethnic groups [19,35]. Furthermore, several studies have shown a greater risk of stroke in women and younger people in the US population [32,36]. Several population-based studies suggested that young individuals with HIV infection have an increased risk of stroke compared to non-HIV individuals, suggesting the role of HIV infection as a cerebrovascular risk factor [17,37,38]. Reduction of viral load in the brain and early assessment of other risk factors of stroke in HIV patients could be one of the preventive measures. However, stroke prevention could be complicated by interaction with ART treatments [23]. The metabolic complications of specific ART approaches have led to the search for alternative agents with better metabolic profiles. However, inability of other ART agents to cross the BBB limits their utility. Extracellular vesicles (EVs) are emerging as a potential marker for Transient Ischemic Attack and also as a therapy for ischemic stroke [39,40,41]. Further, EVs are gaining importance as drug carriers due their immunogenicity, low toxicity and biodegradability and ability to cross the blood–brain barrier (BBB). Therefore, loading EVs with ART can improve the ART levels in the brain leading to reduction in the viral load and thus reduces the risk of stroke in HIV subjects.

In this article, we will review available data on the association between HIV infection and risk of stroke, based on published clinical and postmortem studies, the possible mechanisms of stroke occurrence in HIV-infected patients, and the approaches to improve stroke outcomes in HIV infected patients.

## 2. Factors Contributing to the Development of Stroke in HIV Infected Subjects

The prevalence of HIV-associated neurovascular complications is increasing in the current era of ART. HIV can remain quiescent in multiple cells of the body including those of the central nervous system (CNS) [42]. In the CNS, viral reservoirs are being identified in astrocytes, microglia, perivascular macrophages, and pericytes [9]. Although ART drugs can prevent new cells from being infected, the cells already harboring HIV viral genome can significantly contribute to the synthesis of toxic viral proteins [43]. These patients exhibit a large number of comorbidities, including neurovascular diseases. However, the molecular mechanisms behind the development of such co-morbidities remain elusive. In addition to other factors, the contribution of residual viral protein and toxicity of ART drugs cannot be ignored. Persistent activity of viral protein in HIV infected immune-suppressed patients can induce tissue inflammation, cell death, and activation of aberrant signaling pathways. Additionally, the existence of the blood-brain barrier (BBB) limits drug penetration and makes the brain a viral reservoir [9].

Multiple factors, linked to the HIV infection, could increase the susceptibility of developing cerebrovascular diseases (CVD), such as opportunistic infections, coagulation abnormalities, dyslipidemia, and toxicity of ART. HIV is thought to contribute to the incidence of stroke through both HIV-associated and traditional stroke risk factors. Although ART has revolutionized the life expectancy of HIV infected subjects, the prevalence of age-related diseases has increased as a consequence [44]. Factors contributing to the development of stroke in HIV infected subjects are summarized in Table 1 and discussed below.

### 2.1. Coagulopathies

HIV is associated with various coagulopathies such as thrombotic thrombocytopenic purpura, proteins S and C deficiency, and anti-phospholipid syndrome [45,46,47]. Proteins S and C are potassium-dependent anticoagulants. Decreased levels or impaired function of proteins S and C leads to a propensity for venous thrombosis. A case-controlled study in HIV infected patients with ischemic stroke has shown that 45% of patients had protein S deficiency [46]. In contrast, a study by Mochan and colleagues showed no significant association between protein S deficiency and the occurrence of stroke in HIV positive patients, suggesting that protein S deficiency is an epiphenomenon of the HIV infection with no recognized relationship to stroke [96]. Although deficiencies in protein C and protein S were observed in stroke patients with HIV, the question remains unclear whether these deficiencies are secondary events or directly caused by HIV infection. Additionally, a case-controlled study in young HIV positive stroke patients demonstrated that elevated levels of VWF in comparison with both uninfected and HIV-infected patients without stroke group [97]. These responses could be mediated by HIV-induced endothelial dysfunction which may induce inflammation and prothrombotic state.

### 2.2. Opportunistic Infections

Certain opportunistic infections including tuberculosis meningitis, neurosyphilis, and varicella-zoster vasculitis may predispose an individual to ischemic stroke. *Tubercle bacillus* (TB) is the most common pathogen known to cause opportunistic infections associated with HIV. In fact, stroke is thought to be a complication of tuberculous meningitis [50]. The HIV infection results in an immunocompromised state that increases one’s susceptibility for secondary infections [51]. Varicella zoster infection also increases the risk of stroke and cerebral vasculitis in immunosuppressed patients [52]. Increased meningovascular complications and neurosyphilis have been observed in people with HIV [53]. These infections are thought to induce widespread neurovascular inflammation leading to endarteritis and a prothrombotic state. The combination of inflamed arterial walls with a predisposition to thrombus formation contributes to increased risk for atherosclerosis which leads to an ischemic stroke [10]. Although *Candida albicans* and cytomegalovirus infections have been associated with HIV infection and stroke in a few case reports, further investigation is required to confirm their roles in the pathogenesis of ischemic stroke in HIV cases [54,55].

### 2.3. HIV Associated Vasculopathy

Despite an advancement in the ART, vascular disease has appeared as a major root of morbidity and mortality in HIV infected population and is the leading cause of stroke [98]. Data obtained from post-mortem human brain samples demonstrated the evidence of vasculopathy in immunosuppressed patients [44,57]. Systemic analysis of the brain arteries from autopsy samples showed that HIV patients have higher arterial inflammation, more predominantly in the adventitial intima [55]. In addition, atherosclerosis of the large arteries and small vessels is the most common cause of ischemic stroke in HIV infected patients [46,99]. Brilla et al. demonstrated that HIV-infection is associated with reduced baseline blood flow and cerebrovascular reserve capacity [58]. Large vessel vasculopathy with ectasia and aneurysm formation was also observed intra- and extracranially and was associated with intra cerebral hemorrhage and ischemic stroke [59,60].

### 2.4. Cardioembolism

Cardioembolism represents approximately 4-20 % of ischemic strokes in people with HIV [30,46]. The etiology of cardioembolic strokes includes arrhythmias, cardiac chamber abnormalities, and valve disorders. HIV patients have been shown to have a higher risk of atrial fibrillation [60] and are also known to develop cardiomyopathy [62]. Additionally, bacterial and marantic endocarditis, and ischemic heart disease significantly contribute to cardioembolism [63].

### 2.5. Atherosclerosis

HIV infection induces vascular abnormalities such as increased carotid intimal thickness, vascular inflammation, carotid arterial wall stiffness, and abnormalities in vascular compliance and distensibility in the absence of ART, which are major risk factors for atherosclerotic disease [66,67,68]. ART treatment modulates the markers of inflammation, immune activation and coagulation [69]. HIV may directly initiate atherogenesis through activating immune cells and endothelial cells, increasing the numbers of circulating atherogenic immune cells, and modification of lipid levels and function [23].

HIV-associated inflammation plays a significant role in atherosclerosis [70]. The vascular endothelium is constantly exposed to stimuli such as HIV-infected cells (CD4+ T cells, monocytes, macrophages, and circulating viruses), viral proteins that are released with host cell lysis and actively secreted, and viral-induced proinflammatory mediators [71]. These stimuli potentially damage the endothelium and increase its permeability resulting in the extravasation of immune cells, which ultimately results in chronic inflammation [72]. HIV infection induces the synthesis of oxidative free radicals, cell adhesion molecules (CAMs), and release of chemoattractant such as chemokine ligand 2 (CCL2) at the site of inflammation, which attracts leukocytes [73]. In addition, endothelial specific coagulatory molecules such as von Willebrand factor (VWF), thrombomodulin, plasminogen activator inhibitor-1 antigen, tissue factor, and d-dimer are disturbed in HIV infection, favoring a prothrombotic state, which potentially accelerates atherosclerosis [74].

The HIV viral proteins, the trans-activator of transcription (tat), glycoprotein-120 (gp120), and negative regulatory factor (nef) are important contributors to immune activation. The tat and gp120 proteins induce oxidative stress and increase the expression of CAMs and enable transmigration and adhesion of leukocytes to the endothelium [75,76]. The tat induces the expression of monocyte chemoattractant protein-1 (MCP-1), which attracts monocytes to the site of infection and induces the synthesis of tumor necrosis factor alpha (TNF-α), nuclear factor kappa- B (NF-κB) and interleukin-6 (IL-6) [77,78]. The gp120 boosts synthesis of TNF-α and secretion of immunoglobulins in B-lymphocytes. It also induces macrophage activation along with Nef [79,80]. Further, Nef proteins may facilitate the transformation of macrophages into foam cells, which provide the basis for atherosclerotic transformation [81]. Altogether, HIV viral proteins create a pro-inflammatory milieu that facilitates atherogenesis.

Infact, HIV infected patients had more extensive atherosclerosis as measured by Carotid intima-media thickness (cIMT), is a validated measure of subclinical atherosclerosis. Although ART could fully suppress the viral titer, these patients still had higher cIMT than HIV-negative controls [82]. 

### 2.6. Antiretroviral Therapy

It was believed that the elevated incidence of stroke in HIV infected patients was caused by the increased prevalence of opportunistic infections and inflammation due to immunosuppression, and older ART regimens that predisposed patients to dyslipidemia and lipodystrophy [11]. However, ART also contributes to the risk of stroke, both directly by accelerating atherosclerosis and indirectly by increasing life expectancy [10]. Although ART has revolutionized the HIV treatment outcomes, low level of viral suppression in the CNS reservoirs leading to increased risk of stroke cannot be ignored [33,83]. The international multicohort Data Collection on Adverse Events of Anti-HIV Drugs; DAD study has demonstrated that prolonged ART treatment was associated with an increased prevalence of cardiovascular and cerebrovascular disease [83].

Several studies underlined that immunosuppression and high viral loads are associated with increased incidence of stroke [10,31,84]. Consistently, patients with more immunocompetency had a lower frequency of ischemic stroke [85]. Continuous exposure of low viral titer can induce low grade systemic inflammation, which may further add to the risk of stroke [10,86]. Several classes of ART drugs have shown their potential to increase the risk of stroke by inducing endothelial toxicity and vascular dysfunction in HIV [87,88]. Prolonged use of protease inhibitors (PIs) such as darunavir has shown their potential to induce stroke and myocardial infarction [89]. Additionally, atazanavir was shown to be associated with vascular remodeling [90]. Nucleoside reverse transcriptase inhibitor (NRTI), abacavir has also demonstrated an association with increased incidence of cardiovascular events and stroke [91]. Although ART could reduce the virulence of HIV and increased life expectancy in individuals with HIV, with long-term endothelial and metabolic challenges there is an increasing the risk of stroke. Conversely, there are few studies reported that ART is associated with reduced risk of stroke [32,100]. This is where ART functions to control the HIV infection, whereby the viral suppression, and improved immune function confers protection against stroke. However, prolonged exposure to ART may lead to a rise in vascular risk over time.

### 2.7. Traditional Risk Factors

HIV positive patients display a greater occurrence of risk factors for strokes, including hypertension, dyslipidemia, diabetes, coronary artery disease (CAD), and atrial fibrillation [6,61,93,94]. This increases further with age and the improved life expectancy resulting from ART [95,101].

## 3. Experimental Studies on HIV-Associated Ischemic Stroke

Experimental studies in murine models of stroke demonstrated that HIV infection increases the infarct volume [9,102]. HIV infection induces BBB disruption, evidenced by reduced level of tight junction proteins, which enhances BBB permeability and worsen small vessel disease. It can lead to an increase in vascular inflammation, emphasized by overexpression of cell adhesion molecules (CAM) and matrix metalloproteinases (MMPs), as well as infiltration of brain tissue with inflammatory cells [9]. Increased extravasation of neutrophil, microglia, monocytes, and macrophages are originated from brain and peripheral infiltration, which enhances viral overload-induced delayed post stroke recovery.

Although chronic ART is indispensable for maintaining the health of HIV infected patients, serious systemic and local side effects of many classes of ART drugs cannot be ignored [103]. Bertrand et al. demonstrated that efavirenz, a non-nucleoside reverse transcriptase inhibitor (NNRTI), increases the BBB permeability and stroke severity in comparison with other NNRTIs such as etravirine, nevirapine, and rilpivirine [102]. Efavirenz significantly decreased the levels of claudin-5, a transmembrane tight junction protein in primary human cerebral microvascular endothelial cell (hCMEC) monolayers through elevation of endoplasmic reticulum stress (ER stress). Elevation of ER stress is linked to BBB disruption [104]. Conversely, efavirenz treatment in EcoHIV/NDK (a mouse adapted strain of HIV, where gp120 is replaced by gp80) infected mouse did not reduce the expression of claudin-5 in micro vessels. Nonetheless, treatment with efavirenz in EcoHIV/NDK-infected brain reduced expression of Zonula occludens-1 (ZO-1) in brain micro vessels independent of HIV infection [102].

The BBB is an active interface between the CNS and peripheral circulation. It controls the transport of biological molecules required for neuronal function and integrity [105]. Microvascular endothelial cells surrounded by astrocytes and pericytes cooperatively form the BBB, which are held 50-100 times tighter than peripheral micro vessels by means of tight junctions. Disruption of the BBB is one of the pathological features of ischemic stroke, which contributes to progression of brain injury and consequent neurological impairment [106]. MMPs along with endogenous tissue inhibitors of MMPs (TIMS), play a significant role in BBB remodeling. An imbalance between MMPs (MMP-2, MMP-9) and TIMS (TIMP-1, TIMP-2) in peripheral blood as well as in the CSF has been reported in HIV patients with HAND and contributes to the HIV-associated BBB damage [107]. Activated monocytes and macrophages play a significant role in this event in HIV subjects [108]. These activated immune cells enhance the synthesis of MMPs and promotes the migration of viral-infected cells and inflammatory cells into the CNS and accelerate synthesis of inflammatory mediators and migration of toxic substances to the brain. In addition, plasma isolated from HIV subjects could compromise the integrity of in- vitro model of BBB composed of astrocytes and microvascular endothelial cells and enhanced the transmigration of monocytes and macrophages [107]. Further, pharmacological inhibition of MMPs ameliorated BBB damage induced by HIV infection, confirming contributory role of MMPs [109].

C-C chemokine receptor type 5 (CCR5) plays a critical role in HIV infection and cell to cell transmission. CCR5 belongs to chemokine receptor, act as coreceptor for HIV entry into the cells [110]. Recently Joy et al. demonstrated that expression of CCR5 was elevated in cortical neurons after ischemic stroke and neuron-specific knockdown of CCR5 promoted early recovery of motor function following ischemic stroke in mice [111]. Interestingly pharmacological inhibition of CCR5 with maraviroc, an FDA approved antiretroviral drug could improve the motor recovery and neuronal connectivity in a mouse model of ischemic stroke. However, translational potential of this drug in HIV associated stroke has not been evaluated.

## 4. Approaches to Improve Ischemic Stroke Outcomes in HIV Infected Subjects

Although there is a lack of clear mechanistic target for stroke management in HIV-infected individuals, the acute treatment of stroke should be similar to that of the uninfected general population, in accordance with standard guidelines. Early assessment of cause and risk factors of stroke in HIV patients could be one of the preventive measures. However, stroke prevention could be complicated by interaction with ART treatments [23]. The metabolic complications of specific ART approaches have led to the search for alternative agents with better metabolic profiles. In this regard, metabolic complication could be mitigated with combinational approaches of ART with statins or fibrates [112]. Moreover, there is a need of ART without any endothelial and metabolic effect. Tissue plasminogen activator (tPA) is the only FDA approved thrombolytic agent for ischemic stroke. A retrospective review of tPA-treated HIV patients with acute stroke in demonstrated that tPA did not cause any complications or fatalities in HIV patients [113], implies that no risk of hemorrhage in HIV infected patients with stroke if they receive thrombolytic therapy. In addition, drug such as dabigatrin (an anticoagulant) and aspirin (antiplatelet agents) have shown no specific drug interaction with ART [23]. In the following section, we are discussing the possible novel approaches for the management of stroke in HIV infected patients.

### 4.1. ART With a High CNS Penetration Efficacy (CPE)Score

HIV infection increases the risk of stroke through various mechanisms as described earlier [10]. Once a stroke is diagnosed, the goal should be directed towards acute stroke management, establishment of the cause of the stroke, management of HIV infection, and secondary prevention of stroke [112]. Ultimately, continuing antiretroviral therapy without the metabolic and endothelial effects and keeping viral load undetectable help reduce the risk of ischemic stroke [10]. Though the ART drugs suppress peripheral viral load, they cannot efficiently eliminate the virus from the CNS due to their low CNS bioavailability. In an experimental ischemic models, Bertrand et al. showed that an ART combination with high CNS penetration-effectiveness (CPE) score (zidovudine, emtricitabine, and nevirapine) significantly decreased the infract size and accelerated post-stroke recovery compared to an ART combination (raltegravir, emtricitabine, and tenofovir), with a low CPE score [9]. Thus, ART drugs with high CPE scores could suppress the virus and reduce the risk of stroke in HIV infected subjects. However, Data Collection on Adverse Events of Anti-HIV Drugs (D:A:D) study looked at the effect of 3 classes of ART drugs NRTIs (zidovudine, stavudine, didanosine, zalcitabine, lamivudine, abacavir, and tenofovir), 4 PIs (indinavir, nelfinavir, lopinavir-ritonavir, and saquinavir), and 2 NNRTIs (efavirenz and nevirapine) on the risk of myocardial infarction in HIV subjects. They reported that only indinavir, lopinavir-ritonavir, abacavir, and didanosine were associated with a significantly increased risk of myocardial infarction, and stroke [114]. Hence, careful selection of ART drugs should be made to treat HIV positive subjects who also have other co-existing risk factors such as cardiovascular and metabolic disorders or TB and other infections [115]. Furthermore, effective ART with a high CPE scores should be developed to treat stroke in HIV infected subjects.

### 4.2. Tat Fusion Protein

HIV-Tat protein was shown to cross the cell membranes [116]. Interestingly, few larger peptides, which are linked to HIV-Tat, have also been shown to cross the cell membranes, but their exact mechanism is still a matter of debate [117]. Intravenous injection of anti-apoptotic (Bcl-XL) and neurotrophic (GDNF) factors fused with HIV-Tat protein significantly reduced brain injury in mice subjected to focal cerebral ischemia [117]. Since HIV infected subjects already have the Tat protein in their system, directing Bcl-XL and GDNF to Tat protein in the body would probably reduce chances of ischemic stroke in those subjects.

### 4.3. Extracellular Vesicles as Carriers

Extracellular Vehicles (EVs); the nanosized non replicative (≤200nm) lipid membrane-bound vesicle are mainly involved in the intercellular communication [118]. EVs are secreted by almost every cell in the body by the endosomal pathway. In the previous few decades, tremendous research has been done to reveal the possible use of EVs as biomarkers and diagnostic tools for various diseases such as cancer, HIV, arthritis, neurodegenerative disorders, and stroke [119,120,121]. For instance, HIV infected patients have shown a surge in the number of Extracellular Vesicles as compared to healthy individuals [122]. There are primarily two ways to target the HIV-associated stroke, i.e., diagnosis and therapy.

#### 4.3.1. EVs: Ischemic Stroke Diagnosis

As EVs are widely distributed in various body fluids such as blood, urine, and milk; this makes EVs potential biomarkers [123]. EVs are the carriers of various cellular proteins and nucleic acid, which reflect the pathophysiological state of the parent cells. Among all the cargos, micro RNAs (miRNAs) are the entities which are very well studied because of their stability. RT-qPCR is considered the gold standard for measurement of miRNA in the blood of ischemic patients [124]. The EVs present in the plasma of transient ischemic attack (TIA) patient was found to have different expression levels of rno-miR-122-5p and rno-miR-300-3p [41]. According to a clinical study, miR-223 is strongly linked with ischemic stroke and a potential biomarker [125]. Another clinical study performed on 65 IS patients showed that, the level of miR-9 and miR-124 were related to National Institutes of Health Stroke Scale (NIHSS) scores [126]. As the miRNAs present in the EVs are protected from the degradation and are stable, this makes the EVs a novel candidate for liquid-based biomarkers. In addition, the physical characteristics such as EVs size, and concentration are also an important parameter, which are generally elevated in the disease conditions [120,127].

#### 4.3.2. EVs: Ischemic Stroke Therapy

**Figure 1:**
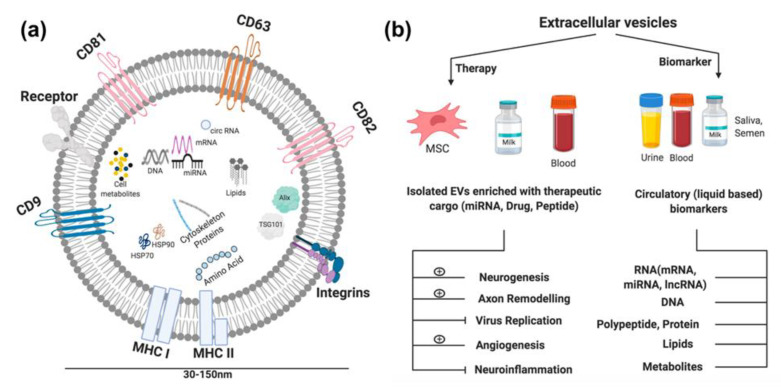
Extracellular vesicles and their use as therapeutic and diagnostic tools. (**a**) Extracellular vesicles are nano-sized vesicles produced by endocytic pathway. EVs carry various types of cargo molecules, such as, nucleic acids (RNA, DNA, miRNA), proteins, metabolites and lipids. EVs carries signal from one part to another part of body. (**b**) EVs carries signals from one part of the body to another. EVs can be used as potential therapeutics and biomarkers for HIV related stroke. Therapy: EVs could be derived from various sources such as MSC (mesenchymal stem cells), and other body fluids; milk and blood (serum/plasma). These EVs could be fortified with other molecules; miRNA, drugs or peptides, which could promote or inhibit various factors related to HIV replication and stroke. Diagnosis: The contents of EVs changes as per the different states of disease manifestations thus can be used as biomarkers.

The main treatment of stroke is to restore blood flow in the respective blood vessels as soon as it is diagnosed. EVs are highly conserved among most of organisms [128]. As EVs are responsible for intercellular communication, they could potentially be used as a novel treatment strategy. Over the past several decades, ample amount of research has been conducted on the application of EVs as therapies for various diseases [119,120,121,129]. Although, ultracentrifugation is considered the gold standard for EV isolation, now-a-days various commercially available reagents are available, which can isolate EVs of high purity, with great yield [130]. Some commercially available kits rely on different principles such as precipitation methods or size exclusion chromatography (SEC) [131,132]. As stability of the carrier is an important measure to develop any new therapy; EVs are stable up to one week at 4 °C and could be stored for up to 3 months at -80°C [133]. EVs are also reported to cross the BBB [134]. As EVs contain the cell specific ligand it can be used as targeted therapy. Modified EVs could be used as transporters of various entities such endogenous gene, proteins, and even drugs to the target cells. Stricture of the EV and its application is depicted in Figure 1. *In vivo* study reports that, mesenchymal stromal cells (MSC)-derived EVs when injected intravenously, improved cell recovery post stroke [135]. The MSC-derived EVs were fortified with miR-17-92 clusters and had enhanced functional recovery and neural plasticity via PI3K/Akt/mTOR/GSK-3β pathway [136]. Further, MSCs and EV based therapy have shown promising results in the early phase of clinical trials for cancers and strokes [137,138]. Table 2 summarizes the studies on application of exosomes/ EVs in HIV and stroke.

Nevertheless, some challenge must be overcome before EVs can be used in clinical trials. These are: (1) Current EV strategies are of small scale, there is a need to develop large scale technologies to promote rapid and efficient isolation s; (2) As endogenous EVs can cross BBB, the modification of their surface characteristics could be a promising way to enhance the accuracy of drug delivery [139]; (3) Even though, various commercial methods are available for isolating EVs with high purity, they have some limitations such as use of very expensive equipment and the requirement of large sample volumes.

Nevertheless, some challenge must be overcome before EVs can be used in clinical trials. These are: (1) Current EV strategies are of small scale, there is a need to develop large scale technologies to promote rapid and efficient isolation s; (2) As endogenous EVs can cross BBB, the modification of their surface characteristics could be a promising way to enhance the accuracy of drug delivery [135]; (3) Even though, various commercial methods are available for isolating EVs with high purity, they have some limitations such as use of very expensive equipment and the requirement of large sample volumes.

## 5. Conclusions and Future Directions

Multiple challenges exist when dealing with the clinical manifestation of HIV-associated stroke. Improved life expectancy in treated HIV patients increases incidence traditional risk factors along with prolonged viral infection and side effects of ART. There are various ways to confront these challenges, starting from preventive measures to early screening of risk factors and novel therapeutic interventions. Recent advances in stroke reperfusion therapies have led to remarkable improvement in clinical outcomes, however, a small population of patients are gaining benefit due its narrow therapeutic window. ART drugs are known to be beneficial for suppressing the viral load, but their prolonged use is still not clinically proven safe for the HIV patients. Nevertheless, its side effects are overweighed by extensive benefits, addressing the need to develop modified ART with minimal adverse effect and better HIV control. Additionally, there is an urgent need to develop novel approaches, that might provide new opportunities for stroke treatment. In recent years the clinical potential of exosomes for stroke diagnosis and therapy has attracted widespread attention due to their unique characteristics. However, there is a lack information to translate the exosome therapy into clinical practice. Further, challenges associated with use of EVs in clinical practice such as on a large-scale synthesis of EVs at a lower cost, lack of isolation techniques to obtain pure EVs. Further investigations are warranted to elucidate molecular mechanisms mediated by exosome and the generation of clinical-grade exosomes for seamless clinical translation.

## Figures and Tables

**Table 1:** Summary of factors contributing to the prevalence of ischemic stroke in HIV patients.

Risk Factors	Causes	Effect on Stroke	Reference
Coagulopathies	Thrombocytopenia purpura Protein S & C deficiency Elevated von willebrand factor (VWF) increment in antiphospholipid antibody titres, increase in D-dimer level, microbial translocation, altered platelet morphology	Platelet activation Inflammation Endothelial activation Venous thrombosis.	[45,46,47,48,49]
Opportunistic infections	*Mycobacterium tuberculosis*, neurosyphilis, *Candida albicans,* cytomegalovirus, varicella-zoster	Neurovascular inflammation leading to endarteritis and a prothrombotic state vasculitis and endarteritis elevated meningovascular complications	[50,51,52,53,54,55,56]
HIV-associated vasculopathy	Intracranial or extracranial cerebral abnormality of the blood vessels (etacia and aneurism) arterial inflammation in the adventitial intima	Vascular inflammation atheroschlerosis reduced cerebral blood flow and cerebrovascular reserve capacity	[44,57,58,59,60]
Cardioembolism	Opportunistic infections including bacterial endocarditis valvular disorders cardiac chamber abnormalities dilated cardiomyopathy, ischemic heart diseases	Atrial fibrillation	[61,62,63,64,65]
Atherosclerosis	Increased carotid intimal thickness (cIMT), vascular inflammation, abnormalities in vascular compliance, activation of immune cells Elevated release of pro-inflammatory mediators by viral proteins Increased oxidative stress, chemo attractants (eg: CCL2), cell adhesion molecule (CAM) elevated endothelial specific coagulatoty molecules	Immune activation, vascular inflammation, endothelial activation, development of atherosclerotic plaques	[23,66,67,68,69,70,71,72,73,74,75,76,77,78,79,80,81,82]
Antiretroviral therapy	Endothelial toxicity, low grade systemic inflammation, dyslipidemia and vascular dysfunction, enhancement of large-vessel atherosclerosis	Vascular dysfunction, atherosclerosis, myocardial infarction and cerebrovascular diseases	[10,11,83,84,85,86,87,88,89,90,91,92]
Traditional risk factors	Hypertension, dyslipidemia, diabetes, coronary artery disease (CAD) and atrial fibrillation	Hypertension, diabetes can lead to chronic inflammation myocardial remodeling, and atrial fibrillation likelihood of large-vessel atherosclerosis	[6,61,93,94,95]

**Table 2:** Extracellular vesicles/exosomes derived from various sources and their effect on HIV and stroke.

Sources of EVs/Exosomes	Study Mode	Effector Molecule/Component	Outcome	References
Rat bone marrow-derived mesenchymal stem cells derived extracellular vesicles	In vivo	miRNA-17–92	Increase neural plasticity and functional recovery after stroke	[136]
Human semen exosome	In vitro and In vivo	mRNA	Inhibit intravaginal transmission and proliferation of HIV complex.	[140,141]
Human milk exosomes	In vitro	Mucin 1	Inhibit the vertical transmission of HIV to monocyte-derived dendritic cells	[142]
Cell culture supernatants of HIV-1-infected cells and HIV-1- patient serum derived exosomes	In vitro	trans-activation response element (TAR) miRNA	Promote HIV infection	[143]
Cell culture exosomes and Microvesicles	In vitro	immune response factors, adhesion and viral proteins	Facilitate HIV-1 infection	[144]
Cell culture exosomes	In vivo	miR-133b	Improve neural plasticity and functional recovery after stroke	[145]
Rat adipose-derived mesenchymal stem cells exosomes	In vivo	Proteins	Improve functional recovery, axonal sprouting and white matter repair fiber tract integrity	[146]
Rat bone marrow-derived mesenchymal stem cells derived extracellular vesicles.	In vivo	MiRNA-17–92	Increase neural plasticity and functional recovery after stroke	[136]

## Abbreviations

AIDS	Acquired immunodeficiency syndrome
ART	Antiretroviral therapy
BBB	Blood-brain barrier
CAMS	Cell adhesion molecules
CCCR-5	C:C chemokine receptor type 5
CCL2	Chemokine ligand 2
cIMT	Carotid intima-media thickness
CNS	Central nervous system
DAD	Data Collection on Adverse Events of Anti:HIV Drugs
ER stress	Endoplasmic reticulum stress
EVs	Extracellular vesicles
Gp120	Glycoprotein-120
hCMEC	human cerebral microvascular cells
HIV	Human immunodeficiency virus
IL-6	Interleukin-6
MCP-1	Monocyte chemoattractant protein-1
miRNA	Micro RNA
MMP	Matrix metalloproteinases
MSC	Mesenchymal stem cells
Nef	Negative regulatory factor
NF:κB	Nuclear factor kappa-B
NNRTI	Reverse transcriptase inhibitor
PLWH	People living with HIV
SEC	Size exclusion chromatograohy
Tat	Trans-Activator of Transcription
TB	*Tubercle bacillus*
TIA	Transient ischemic attack
TIMs	Tissue inhibitors of MMPs
VWF	von Willebrand factor
ZO-1	Zonula occludens-1
